# Impaired glucose metabolism in subjects with the Williams-Beuren syndrome: A five-year follow-up cohort study

**DOI:** 10.1371/journal.pone.0185371

**Published:** 2017-10-20

**Authors:** Maria Elena Lunati, Maria Francesca Bedeschi, Veronica Resi, Valeria Grancini, Eva Palmieri, Simona Salera, Faustina Lalatta, Giuseppe Pugliese, Emanuela Orsi

**Affiliations:** 1 Diabetes Service, Unit of Endocrinology and Metabolic Diseases, IRCCS “Cà Granda—Ospedale Maggiore Policlinico” Foundation, and Department of Medical Sciences, University of Milan, Milan, Italy; 2 Unit of Medical Genetics, IRCCS “Cà Granda—Ospedale Maggiore Policlinico” Foundation, Milan, Italy; 3 Direzione Sanitaria di Presidio, “Cà Granda—Ospedale Maggiore Policlinico” Foundation, Milan, Italy; 4 Diabetes Unit, Sant’Andrea Hospital, and Department of Clinical and Molecular Medicine, “La Sapienza” University, Rome, Italy; University of Michigan, UNITED STATES

## Abstract

**Objective:**

The Williams-Beuren syndrome (WS) is associated with impaired glucose metabolism (IGM) early in adulthood. However, the pathophysiology of IGM remains poorly defined, due to the lack of longitudinal studies investigating the contribution of β-cell dysfunction and impaired insulin sensitivity. This study aimed at assessing incidence of IGM and the underlying mechanisms in WS adults.

**Methods:**

This observational, longitudinal (5-year), cohort study enrolled thirty-one consecutive WS subjects attending a tertiary referral center. An oral glucose tolerance test (OGTT) was performed yearly and used to classify patients as normal or IGM, including impaired fasting glucose (IFG) and/or impaired glucose tolerance (IGT) and diabetes mellitus (DM), and to calculate surrogate measures of insulin secretion and/or sensitivity.

**Results:**

IGM patients were 18 (58.1%, three DM) at baseline and 19 (61.3%, five DM) at end-of-follow-up. However, 13 individuals changed category of glucose homeostasis in both directions during follow-up (8 progressors, 5 regressors) and 18 did not (8 non-progressors, 10 non-regressors). New cases of IGM and DM were 11.1 and 2.53 per 100 persons-year, respectively, and were treated non-pharmacologically. In the whole cohort and, to a higher extent, in progressors, indices of early-phase insulin secretion and insulin sensitivity decreased significantly from baseline to end-of-follow-up, with concurrent reduction of the oral disposition index and insulin secretion-sensitivity index-2 (ISSI-2), compensating insulin secretion for the level of insulin resistance. No baseline measure independently predicted progression, which correlated with change from baseline in ISSI-2. Compared with patients with normal glucose homeostasis, IGT subjects had impaired insulin sensitivity, whereas insulin secretion was reduced only in those with IFG+IGT or DM.

**Conclusions:**

IGM incidence is high in young adults with WS, suggesting the need of early screening and timed intervention. As in classical type 2 diabetes, impaired insulin sensitivity and β-cell dysfunction contribute, in this sequence, to progression to IGM and DM.

## Introduction

Several genetic disorders are accompanied by an increased incidence of diabetes mellitus (DM). In these disorders, which are classified among the “other specific types of DM” [1], impaired glucose metabolism (IGM) does not represent the main disease manifestation, at variance with the monogenic forms of DM. In addition, DM is not invariably present in individuals suffering from these syndromes and, when present, it is often diagnosed only by performing an oral glucose tolerance test (OGTT). Though rare, these disorders may provide insight into the pathophysiology of the common forms of DM, especially type 2 DM. In particular, they may shed light on the relative contribution of β-cell dysfunction and impaired insulin sensitivity and the molecular mechanisms of these abnormalities.

Williams-Beuren syndrome (WS; OMIM 194050) is a rare, multi-systemic genomic disorder due to an accidental mis-pairing of chromosome 7 leading to a 1.55–1.8Mb deletion in the q11.23 region (WS chromosome region, WSCR). Deletion causes a loss of 26–28 genes, including the *ELN* gene, which codes for the protein elastin, the lack of which has been associated with the main WS abnormalities [2,3]. Prevalence of this syndrome ranges from 1/7,500 to 1/20,000 [4]. The clinical phenotype is well characterized during infancy and childhood. The main features include the characteristic facial dysmorphisms, growth delay, intellectual disability with typical neurobehavioral profile, and cardiovascular abnormalities, most often supravalvular aortic stenosis and/or peripheral pulmonary stenosis. Arterial hypertension, subclinical hypothyroidism, gastrointestinal disturbances (gastroesophageal reflux, abdominal pain, constipation, and diarrhea), recurrent urinary tract infections, and orthopedic problems have also been described [5–7]. Conversely, few descriptions of clinical features in adults are actually available [5,6]. Adults with WS are typically limited in their ability to live independently or work in competitive employment settings, due to the persistence into adulthood of physical and mental health problems and the emergence of new medical issues such as audiological, dental, and endocrine abnormalities. In particular, these individuals frequently develop IGM, comprising impaired fasting glucose (IFG), impaired glucose tolerance (IGT), and DM, early in the adulthood. Previous cross-sectional surveys reported that IGM, as assessed by an OGTT, was observed in 18 out of 20 (90.0%) [5], 21 out of 28 (75.0%) [8], and 14 out of 22 (63.6%) [9] adult WS subjects (mean age 30–40 years), respectively. These high frequencies suggest a genetic basis for IGM and the implication of hemizygosity for one or more genes of WSCR. In particular, two genes mapping to the WSCR have been considered as possible “diabetogenic hits”, syntaxin-1A (STX-1A) [10] and Max-like protein X interacting protein like (MLXIPL, formerly WSCR 14 and also known as carbohydrate-responsive element-binding protein, ChREBP) [11].

Previous studies failed to demonstrate a dysfunction of β-cells in WS adults [8,9], whereas most of them showed an impairment of insulin sensitivity [5,9], thus pointing to a major role of insulin resistance in the pathogenesis of IGM and DM in these individuals. However, the pathophysiology of IGM in the WS remains poorly defined, due to the lack longitudinal studies investigating the contribution of β-cell dysfunction and impaired insulin sensitivity to the development of these abnormalities.

This study was aimed at assessing (a) the incidence rate of IGM and type 2 DM over a 5-year follow-up in adult subjects with the WS; and (b) the pathophysiological mechanisms underlying alterations of glucose homeostasis in these individuals.

## Subjects and methods

### Design

This is an observational, longitudinal cohort study on the abnormalities of glucose regulation detected in adult WS subjects over a 5-year follow-up (see S1 STROBE Statement).

### Ethics

The research protocol complies with the Declaration of Helsinki.

According to the national laws, ethics approval by the locally appointed Ethics Committee was not required because: no intervention was applied; no identifiable private information was collected; patients underwent only routine diagnostic and therapeutic procedures, according to current guidelines of the American Diabetes Association (ADA) [12]; and an anonymized dataset was analyzed.

Patients or their legally authorized representatives gave written informed consent.

### Participants

Thirty-one WS subjects (aged 27.3±5.6 years; 15 males, 48.4%, males; 7 with family history of DM, 22.6%) who were attending the Unit of Medical Genetics, IRCCS “Cà Granda—Ospedale Maggiore Policlinico” Foundation, in 2011 were included in the study. In all cases, diagnosis of WS had been established in infancy based on the characteristic clinical features and confirmed by the typical elastin gene hemizygosity shown by fluorescence in situ hybridization. The cohort included two pairs of twins. None of the parents had clinical features of the WS suggesting that all cases were de novo presentations.

Patients were evaluated for their metabolic profile at the Diabetes Service, Unit of Endocrinology and Metabolic Diseases, IRCCS “Cà Granda—Ospedale Maggiore Policlinico” Foundation, from 2011 to 2016. All patients received individual nutritional and exercise counseling and those with IGM or DM were treated according to standard care, as defined by the ADA guidelines [12].

### Measurements

Anamnestic, clinical, and laboratory data were obtained from all patients at baseline and every 6 months for 5 years, using a standardized protocol. An OGTT was performed every year to assess glucose homeostasis and to estimate β-cell function and insulin sensitivity by the use of surrogate measures.

#### Demographics, clinical history, and metabolic profile

Study subjects underwent a structured interview to collect the following information: age, family history of DM, known DM onset and duration, co-morbidities, and current treatments.

Body weight and height were measured with scale and stadiometer, and body mass index (BMI) was calculated as weight (kg)/height^2^ (m^2^). Patients were then classified as normal-weight, overweight, or obese according to the World Health Organization criteria [13]. Blood pressure was measured with a sphygmomanometer with the patients seated with the arm at the heart level.

Biochemical tests were centralized at the Laboratory of Clinical Chemistry of the IRCCS “Cà Granda—Ospedale Maggiore Policlinico” Foundation. Hemoglobin A_1c_ (HbA_1c_) was assessed by the use of a high performance liquid chromatography, National Glycohemoglobin Standardization Program-certified and Diabetes Control and Complications Trial-standardized method (VARIANT™ II TURBO Hemoglobin Testing System, Bio-Rad laboratories Srl, Segrate, Italy). Fasting levels of glucose, triglycerides, and total, LDL and HDL cholesterol were measured by standard analytical techniques. Fasting insulin and C-peptide levels were measured by ECLIA methods (Elecsys® 2010, Roche Diagnostics SpA, Milan, Italy). In addition, islet cell antibody (ICA) and glutamic acid decarboxylase antibody (GADA) were assessed by an indirect immunofluorescence kit (Euroimmun, Luebeck, Germany) and a radioimmunoassay kit (RSR limited, Cardiff, Great Britain), respectively, to rule type 1 DM or latent autoimmune diabetes of the adult.

#### Indices of insulin secretion and/or sensitivity

A 2-hour-75 g OGTT was performed in all patients, except one subject who had known type 2 DM at baseline. Samples were obtained before and every 30 minutes after load, for measurement of glucose, insulin, and C-peptide levels, using the methods reported above. Based on baseline and 2-hour glucose levels (and HbA_1c_ values) patients were then classified as having normal fasting glucose (NFG)/normal glucose tolerance (NGT), IFG, IGT, or both, or DM, according to the ADA criteria [12]. Values of glucose, insulin, and C-peptide during OGTT were also used to calculate surrogate measures of insulin secretion and/or sensitivity (see Table 1 for calculations). These measures included:

1. indices of β-cell function under fasting conditions (Homeostasis Model Assessment [HOMA]-β-cell function [B%] [14]) and in response to the OGTT (early-phase insulin / C-peptide response: Insulinogenic Index [IGI] at 30 min [15], and C-peptidogenic Index [CPGI] at 30 min [16]; late-phase insulin response: Corrected Insulin Response [CIR]_120_ [17]);
2. indices of insulin sensitivity under fasting conditions (HOMA-Insulin Resistance [IR] insulin [I] [14], HOMA-IR C-peptide [CP] [16]; and Quantitative Insulin Check Index [QUICKI] [18]) and in response to the OGTT (Composite Insulin Sensitivity Index or Matsuda Index [MI] [19]);
3. overall indices (Oral Disposition Index [oDI] I [20], oDI CP [20], and Insulin Secretion-Sensitivity Index-2 [ISSI-2] [21]).

**Table 1:** Surrogate measures of insulin secretion and/or sensitivity.

Index category	Index name	Calculation	Reference
***Insulin secretion***			
Fasting	HOMA-B%	[I_0_ (mU/L) · 20 / G_0_ (mmol/L)– 3.5]	[14]
Early-phase insulin / C-peptide response	IGI at 30 min	[I_30_ –I_0_ (mU/L)] / [G30 –G_0_ (mg/dl)]	[15]
	CPGI at 30 min	[CP_30_ –CP_0_ (ng/ml)] / [G_30_ –G_0_ (mg/dl)]	[16]
Late-phase insulin response	CIR_120_	[I_120_ (mU/L) / G_120_ (mg/dl)] ∙ [G_120_ (mg/dl)– 70]	[17]
***Insulin sensitivity***			
Fasting	HOMA-IR (I)	[I_0_ (mU/L) ∙ G_0_ (mmol/L) / 22.5]	[14]
	HOMA-IR (CP)	[CP_0_ (ng/ml) ∙ G_0_ (mmol/L) / 22.5]	[16]
	QUICKI	1 / [log G_0_ (mg/dl) + log I_0_ (mU/L)]	[18]
During OGTT	MI	10.000 / √ [G_0_ (mg/dl) ∙ I_0_ (mU/L)] ∙ [G_mean_ (mg/dl) ∙ I_mean_ (mU/L)] ^a^	[19]
***Overall***			
During OGTT	oDI (I)	IGI · MI	[20]
	oDI (CP)	CPGI · MI	[20]
	ISSI-2	[(AUC-I/AUC-G) ∙ MI] ^b^	[21]

OGTT = oral glucose tolerance test; HOMA-B% = Homeostasis Model Assessment-β-cell function; IGI = Insulinogenic Index; CPGI = C-peptidogenic Index; CIR = Corrected Insulin Response; HOMA-IR = HOMA-Insulin Resistance; I = insulin; CP = C-peptide; QUICKI = Quantitative Insulin Check Index; MI = Composite Insulin Sensitivity Index or Matsuda Index; oDI = oral Disposition Index; ISSI-2 = Insulin Secretion-Sensitivity Index-2; AUC = area under the curve.

^a^ Mean G and I values during OGTT

^b^ AUC calculated using the trapezoidal rule.

### Statistical analysis

Data are expressed as mean±SD for continuous variables and as number of cases and percentage for categorical variables.

The incidence rates of IGM and DM were calculated by the formula: number of new cases (based on the yearly OGTT) / sum of person-time at risk, and expressed as cases per 100 person-years.

Based on the results of the OGTT performed at baseline and year 5, patients were then classified as progressors, if they passed from NFG/NGT or IFG and/or IGT to IFG and/or IGT or DM, respectively, or regressors, if they showed the opposite trend. In addition, subjects who were in the NFG/NGT category or in the IGM (IFG and/or IGT or DM) category at both time points were classified as non-progressors and non-regressors, respectively. In the whole cohort and in each of these groups, baseline and end-of-follow-up values were compared using the paired Student’s t test, for parametric continuous variables, or the Wilcoxon signed-rank test, for nonparametric continuous variables. Moreover, the four groups were compared for baseline and end-of-follow-up values and changes from baseline to end-of-follow-up by the use of one-way ANOVA (followed by post hoc Bonferroni correction for multiple comparisons) or the Kruskal-Wallis test in case of parametric and non-parametric distribution, respectively. The same tests were used for comparing the various categories of glucose homeostasis (NFG/NGT, IGT, IFG+IGT, and DM) at baseline and end-of-follow-up. Normality of distribution was preliminary assessed by the Kolmogorov–Smirnov test. A *P*-value <0.05 was considered significant.

Binary logistic regression analysis with backward conditional entering of variables was then applied to identify independent correlate(s) of progression from NFG/NGT to IFG and/or IGT or from IFG and/or IGT to DM. Covariates were age, family history of DM, and the baseline levels or the baseline to-end-of-follow-up changes of calculated indices of insulin secretion and/or sensitivity. Results were expressed as odd ratios (ORs) with their 95% confidence intervals (CIs).

Statistical analysis was performed with the statistical package SPSS for Windows version 20.0 (SPPS Inc. Chicago, IL).

## Results

### Changes in metabolic profile from baseline to end-of-follow-up

The baseline and end-of-follow-up metabolic profiles of WS patients not known for type 2 DM at enrollment (30/31) are shown in Table 2. Over the 5-year follow-up, body weight, BMI, HbA_1c_, fasting glucose, insulin, and C-peptide levels, and lipid profile did not change significantly and remained on average within the normal range, except for BMI. Conversely, several important changes were detected in surrogate measures of insulin secretion and/or sensitivity. Regarding β-cell function, the early-phase indices IGI and CPGI decreased, though reduction in IGI did not achieve statistical significance, whereas no significant variation was observed in the fasting and late-phase indices HOMA-B% and CIR_120_, respectively. Regarding insulin sensitivity, MI decreased significantly, whereas no changes were detected in the fasting indices HOMA-IR and QUICKI. As a result, the overall indices, oDI and ISSI-2, which allow compensating insulin secretion for the prevailing level of insulin resistance, decreased significantly from baseline to end-of-follow-up.

**Table 2:** Metabolic profiles of WS patients not known for type 2 DM (30 out of 31) at baseline and end-of-follow-up.

	Baseline	End-of-follow-up	*P*
**Body weight, Kg**	60.5±13.3	60.8±10.8	0.808
**BMI, kg/m**^**2**^	25.0±4.1	25.2±3.1	0.722
**HbA**_**1c**_**, %**	5.33±0.32	5.22±0.31	0.161
**(nmol/mmol)**	(34.8±3.5)	(33.6±3.4)	
**Fasting glucose, mmol/L**	5.16±0.69	5.17±0.70	0.923
**Fasting insulin, pmol/L**	60.9±29.4	71.7±49.0	0.184
**Fasting C-peptide, nmol/L**	0.70±0.31	0.67±0.21	0.641
**Triglycerides, mmol/L**	0.78±0.31	0.70±0.19	0.180
**Total cholesterol, mmol/L**	4.70±0.78	4.61±1.20	0.649
**HDL cholesterol, mmol/L**	1.65±0.45	1.63±0.32	0.835
**LDL cholesterol, mmol/L**	2.91±0.78	3.00±0.82	0.437
**HOMA-B%**	115.3±56.2	128.1±65.7	0.326
**IGI**	1.47±1.49	1.01±0.54	0.092
**CPGI**	0.103±0.057	0.078±0.033	0.015
**CIR**_**120**_	39.3±30.8	46.3±21.6	0.116
**HOMA-IR (I)**	2.05±1.06	2.49±1.90	0.141
**HOMA-IR (CP)**	0.49±0.24	0.48±0.19	0.738
**QUICKI**	0.35±0.03	0.35±0.04	0.639
**MI**	4.19±2.69	3.12±1.68	0.015
**oDI (I)**	4.79±3.64	3.07±2.25	0.006
**oDI (CP)**	0.39±0.26	0.23±0.19	0.006
**ISSI-2**	1.94±0.85	1.60±0.73	0.018

*P* by paired Student’s t test or the Wilcoxon signed-rank test. WS = Williams-Beuren syndrome; BMI = body mass index; HbA_1c_ = hemoglobin A_1c_; HOMA-B% = Homeostasis Model Assessment-β-cell function; IGI = Insulinogenic Index; CPGI = C-peptidogenic Index; CIR = Corrected Insulin Response; HOMA-IR = HOMA-Insulin Resistance; I = insulin; CP = C-peptide; QUICKI = Quantitative Insulin Check Index; MI = Composite Insulin Sensitivity Index or Matsuda Index; oDI = oral Disposition Index; ISSI-2 = Insulin Secretion-Sensitivity Index-2.

### Changes in category of glucose homeostasis from baseline to end-of-follow-up

At baseline (Table 3), 13 patients (41.9%) fell in the NFG/NGT and 18 (58.1%) in the IGM category. Of those with IGM, none had IFG, 8 had IGT, 7 had IFG+IGT, and 3 had DM, which was classified as type 2 DM based on the absence of ICA and GADA positivity. As mentioned above, of DM subjects, two were newly-diagnosed and did not receive any drug treatment, whereas one had known DM and was already on metformin (2 g/day). Overall, 16 patients (51.6%) were normal-weight, 11 (35.5%) were overweight, and 4 (12.9%) were obese (Table 4). Moreover, 17 out 31 subjects (54.8%) were hypertensive and 15 of them were on anti-hypertensive therapy with one-to-three drugs among β-blockers, calcium-channel blockers, angiotensin converting-enzyme inhibitors, angiotensin II receptor blockers, and thiazide diuretics.

**Table 3:** Changes in glucose homeostasis from baseline to end-of-follow-up.

	Baseline				
	**NFG/NGT**	**IFG**	**IGT**	**IFG+IGT**	**DM**
**Follow-up**	13 (41.9)	0 (0)	8 (25.8)	7 (22.6)	3 (9.7)
**NFG/NGT**					
12 (38.7)	8 (61.5) ^c^	0 (0)	3 (37.5) ^b^	1 (14.3) ^b^	0 (0)
**IFG**					
1 (3.2)	1 (7.7) ^a^	0 (0)	0 (0)	0 (0)	0 (0)
**IGT**					
7 (22.6)	1 (7.7) ^a^	0 (0)	3 (37.5) ^d^	2 (28.6) ^d^	1 (33.3) ^b^
**IFG+IGT**					
6 (19.4)	3 (23.1) ^a^	0 (0)	1 (12.5) ^d^	2 (28.6) ^d^	0 (0)
**DM**					
5 (16.1)	0 (0)	0 (0)	1 (12.5) ^a^	2 (28.6) ^a^	2 (66.7) ^d^

Values are n (%).

^a^ Progressors

^b^ regressors

^c^ non-progressors

^d^ non-regressors.

NFG = normal fasting glucose; NGT = normal glucose tolerance; IFG = impaired fasting glucose; IGT = impaired glucose tolerance; DM = diabetes mellitus.

**Table 4:** Changes in BMI category from baseline to end-of-follow-up.

	Baseline		
	NW	OW	OB
**Follow-up**	16 (51.6)	11 (35.5)	4 (12.9)
**NW**			
14 (45.2)	12 (75.0)	2 (18.2)	0 (0)
**OW**			
15 (48.4)	4 (25.0)	9 (87.8)	2 (50.0)
**OB**			
2 (6.4)	0 (0)	0 (0)	2 (50.0)

Values are n (%). BMI = body mass index; NW = normal-weight; OW = overweight; OB = obesity.

At end-of-follow-up (Table 3), 12 subjects (38.7%) were classified as NFG/NGT and 19 (61.3%) as IGM. Of those with IGM, 1 had IFG, 7 had IGT, 6 had IFG+IGT, and 5 had DM. Moreover, 14 patients (45.2%) were normal-weight, 15 (48.4%) were overweight, and 2 (6.4%) were obese (Table 4).

**Fig 1:**
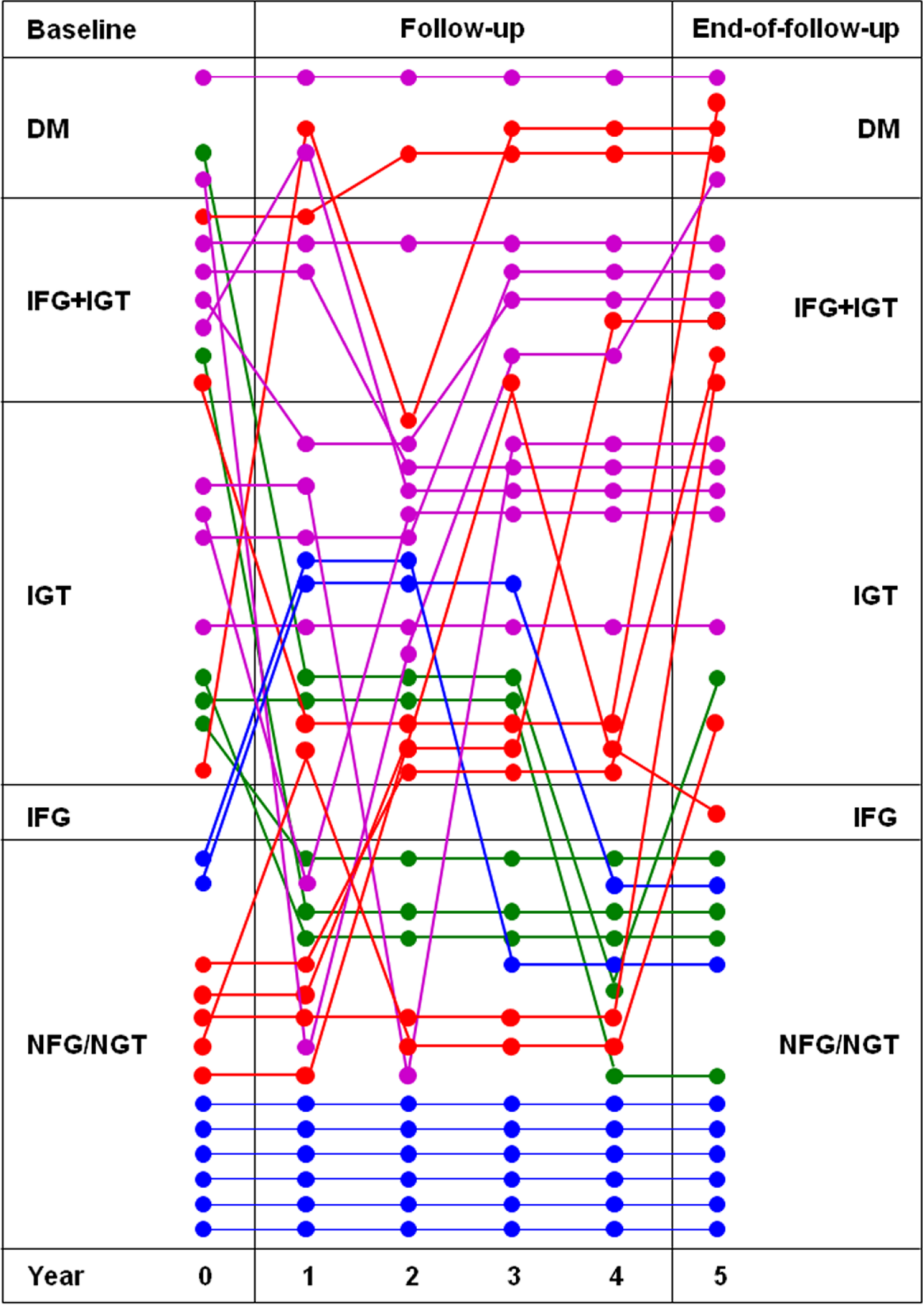
Change of category of glucose homeostasis from baseline to end-of-follow-up in each of the 31 study subjects. Colors indicate progressors (red; n = 8), regressors (green; n = 5), non-progressors (blue; n = 8), and non-regressors (purple; n = 10). NFG = normal fasting glucose; NGT = normal glucose tolerance; IFG = impaired fasting glucose; IGT = impaired glucose tolerance; DM = diabetes mellitus.

Though only one more case of IGM was observed at year 5 (but 2 of DM), as compared with baseline (13 versus 12), 13 individuals (41.9%) changed category of glucose homeostasis from baseline to end-of-follow-up in both directions (Table 3, S1 Table and Fig 1). The incidence rate of new cases of IGM was 11.1 per 100 persons-year and that of DM was 2.53 per 100 persons-year, considering also transient worsening of glucose homeostasis at intermediate OGTTs. Moreover, 4 patients (12.9%) shifted to a higher BMI category (from normal-weight to overweight) and 4 (12.9%) to a lower one (2 from obese to overweight and 2 from overweight to normal-weight), whereas the majority of study subjects (23/31, 74.2%) remained in the same category (12 normal-weight, 9 overweight, and 2 obese).

### Differences among progressors, regressors, non-progressors, and non-regressors

The number of progressors, regressors, non-progressors, and non-regressors were 8, 5, 8, and 10, respectively (Table 3, S1 Table and Fig 1). Of progressors, five subjects passed from NFG/NGT to IFG (one), IGT (one) or IGT+IFG (three) and 3 from IGT (one) or IFG+IGT (two) to DM. All progressors remained in the same BMI category as at baseline, except the one shifting to IFG, who passed from normal-weight to overweight, though this group gained ~4 kg, on average, during the 5-year follow-up period. Of regressors, four patients (three with IGT and one with IFG+IGT) switched to the NFG/NGT category and one from DM to IGT. Only this latter subject shifted to a lower BMI category (from overweight to normal-weight), whereas the other four patients remained in the same category as at baseline, despite the fact that this group lost ~2 kg, on average. Of non-progressors, six subjects remained in the NFG/NGT category for the entire follow-up period, whereas two fell in the IGT category at 2 or 3 intermediate OGTTs. Moreover, one patient shifted from obese to overweight and two from normal-weight to overweight, but average body weight remained the same in this group. Of non-regressors, two had DM (including the one with known DM under metformin therapy who did not undergo the OGTT) and eight had IGT or IFG+IGT both at baseline and end-of-follow-up; of them, one with DM and two IGT showed transient improvements in glucose homeostasis at intermediate OGTTs. One patient shifted from obese to overweight, one from overweight to normal-weight and one from normal-weight to overweight, whereas the remaining seven subjects did not change BMI category during the 5-year follow-up, though this group lost ~2 kg, on average. Of the 7 subjects with family history of diabetes, 2 were progressors (2/8, 25%), 1 was regressor (1/5, 20%), 2 were non-progressors (2/8, 25%), and 2 were non-regressors (2/10, 20%). Interestingly, while the twin sisters were both non-progressors, the twin brothers behaved differently, as one remained NFG/NGT whereas the other progressed to IGT.

**Fig 2:**
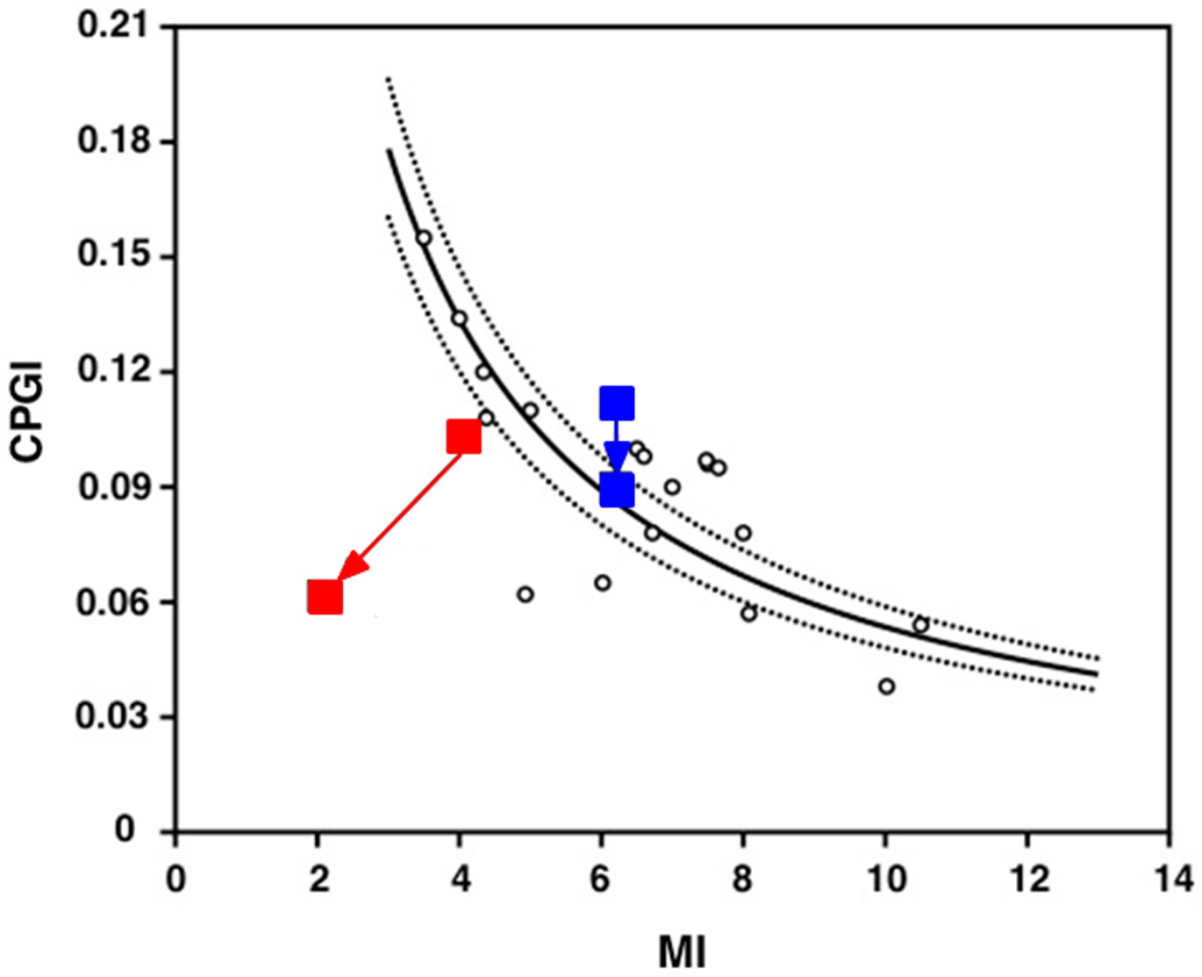
Changes in CPGI relative to changes in MI in progressors (red; n = 8) and non-progressors (blue; n = 8). The lines represent the prediction line and the lower and upper limits of the 95% confidence interval of the regression between CPGI and MI as derived from a reference population with NFG/NGT. CPGI = C-peptidogenic Index; MI = Composite Insulin Sensitivity Index or Matsuda Index; NFG = normal fasting glucose; NGT = normal glucose tolerance.

The metabolic profile at baseline and end-of-follow-up as well as changes from baseline to end-of-follow-up in these parameters did not differ significantly among the four group, except for higher end-of-follow-up values of fasting glucose, and lower end-of-follow-up values of QUICKI, MI, oDI, and ISSI-2 (the latter also at baseline) in progressors than in non-progressors (S1 Fig). Furthermore, while no change from baseline to end-of-follow-up was detected in non-progressors, a significant deterioration of indices of insulin secretion and/or sensitivity was observed in progressors. In particular, there was a significant reduction of CPGI, QUICKI, MI, oDI, and ISSI-2, and an increase of HOMA-IR (I) and CIR_120_, the latter likely reflecting a compensatory increment in late-phase secretion (S1 Fig). As in non-progressors, no change from baseline to end-of-follow-up was observed in both regressors and non-regressors. When plotting the mean value for each group of an index of β-cell function (CPGI) against that of a measure of insulin sensitivity (MI) at baseline and end-of-follow-up. (Fig 2), progressors appeared to loose both insulin secretion and sensitivity with time, whereas non-progressors showed only a slight decrease in secretion. In addition, at baseline, progressors fell below the 95% confidence interval, due to a lower level of insulin sensitivity than that of non-progressors, though differences were not significant (see above).

Binary logistic regression analysis showed that no baseline variable, including family history of diabetes, BMI, and indices of insulin secretion and/or sensitivity, predicted progression from NFG/NGT to IFG and/or IGT or from IFG and/or IGT to DM. Baseline to end-of-follow-up changes in ISSI-2 was the only surrogate measure of insulin secretion and sensitivity correlating significantly with this unfavorable outcome [OR 11.583 (95% CI 1.299–104.279), P = 0.48].

### Contribution of β-cell dysfunction and impaired insulin secretion to abnormalities in glucose homeostasis

Both at baseline and end-of-follow-up, patients with IGT had reduced insulin sensitivity, whereas those with IFG+IGT and DM showed progressively lower indices of first-phase insulin secretion, oDI, and ISSI-2, as compared with NFG/NGT subjects.

**Fig 3:**
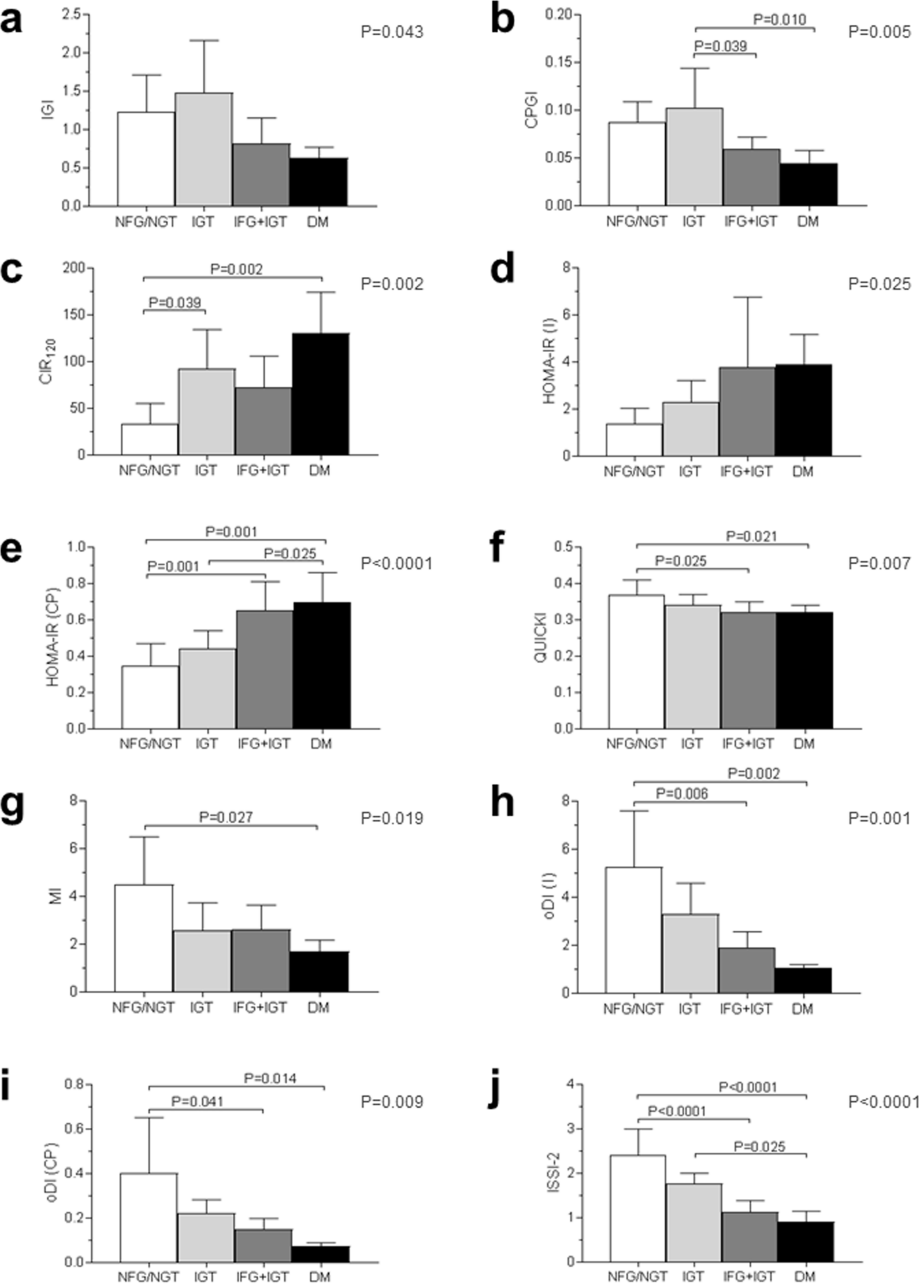
**End-of-follow-up values of IGI (a), CPGI (b), CIR**_**120**_ **(c), HOMA-IR (I) (d), HOMA-IR (CP) (*e*), QUICKI (f), MI (g), oDI (i) (h), oDI (CP) (i), and ISSI-2 (j) in WS subjects with NFG/NGT (n = 12; white bars), IGT (n = 7; light grey bars), IFG+IGT (n = 6; dark grey bars), and DM (n = 4; black bars).** One subject with IFG and one with known DM at baseline (who did not undergo the OGTT) were excluded from this analysis. *P* by ANOVA (right upper end) and *P* by post-hoc Bonferroni correction for multiple comparisons. IGI = Insulinogenic Index; CPGI = C-peptidogenic Index; CIR = Corrected Insulin Response; HOMA-IR = Homeostasis Model Assessment-Insulin Resistance; I = insulin; CP = C-peptide; QUICKI = Quantitative Insulin Check Index; MI = Composite Insulin Sensitivity Index or Matsuda Index; oDI = oral Disposition Index; ISSI-2 = Insulin Secretion-Sensitivity Index-2; NFG = normal fasting glucose; NGT = normal glucose tolerance; IFG = impaired fasting glucose; IGT = impaired glucose tolerance; DM = diabetes mellitus.

Due to the small number (two) of DM subjects with OGTT data at baseline, only end-of-follow-up data are presented (Fig 3).

## Discussion

This is the first prospective study investigating the alterations of glucose homeostasis occurring at a higher rate in adult WS subjects. The results of this survey provide novel information on the epidemiology and pathophysiology of IGM and DM in these individuals.

### New insights into the epidemiology of IGM in the WS

Incidence rate of abnormal glucose regulation, including both IGM and DM, was found to be high in WS patients, considering the young age of participants and the individual counseling intervention. However, though several of these subjects worsened their metabolic status over the 5-year follow-up period, others improved, thus resulting in only one more case of IGM (but two of DM). On the one hand, these findings suggest that alterations of glucose metabolism at this initial stage may still reverse. In this respect, the nutritional and exercise counseling administered to all patients might have been successful in promoting lifestyle changes favoring weight loss and improvement of glucose regulation. On the other hand, failure to comply with lifestyle recommendations by patients who progressed may have resulted in weight gain and driven this unfavorable outcome in susceptible individuals such as those with the WS.

In addition, though longitudinal, this study allowed us to derive data on the prevalence of IGM (and DM), which was also high, consistent with previously published cross-sectional surveys. In particular, the ~60% IGM prevalence observed at baseline and end-of-follow-up is similar to that reported by us in [9] and somewhat lower to the 90% and 75% prevalence values found by other investigators [5,8] in adult WS subjects.

Overall, incidence (and prevalence) data from this study support the concept that WS subjects are at high risk of developing IGM and DM early in adult life and point to the importance of screening these individuals for abnormalities of glucose regulation by an OGTT and providing them with lifestyle recommendations to slow down progression or even favor regression by reducing body weight. Though metabolic abnormalities develop only in the adulthood and their severity appears to be lower than that of other clinical manifestations of WS [5,7], longer follow-up periods are required to establish their rate of progression to overt DM and development of DM complications, which may represent a serious medical problem later in life.

### New insights into the pathophysiology of IGM in the WS

The longitudinal assessment of a wide range of surrogate measures of insulin secretion and/or sensitivity allowed us to demonstrate for the first time that abnormalities of both β-cell function and peripheral insulin action contribute to the impairment of glucose homeostasis and the development of DM in WS patients, as in classical type 2 DM [22].

Our results showed a significant deterioration of indices of first-phase β-cell response, measures of insulin sensitivity, and integrated measures of insulin secretion and sensitivity, such as oDI and ISSI-2, over the 5-year follow-up. Worsening of these indices was detected in the whole cohort, thus indicating that it is a characteristic feature of WS subjects in early adulthood. However, deterioration of glucose regulation was more pronounced in subjects progressing from NFG/NGT to IFG and/or IGT or from IFG and/or IGT to DM, who showed significantly worse values at end-of-follow-up, as compared with baseline. These individuals showed also a trend for an increase in the measures of fasting and late-phase insulin secretion (which was significant for CIR_120_), suggesting an (insufficient) compensatory response to reduction of first-phase insulin secretion. Of the two composite indices of insulin secretion and sensitivity, change in ISSI-2 turned out to be a better correlate of progression in WS subjects than change in oDI, though oDI was previously shown to predict conversion to DM in various populations [23,24]. However, no baseline measure of insulin secretion and/or sensitivity was able to predict progression. While subjects who progressed showed a deterioration of both insulin secretion and sensitivity, those who did not progress exhibited only a mild decrease in secretion.

Also the course of reduced insulin secretion and sensitivity recapitulates the natural history of type 2 DM [25]. In fact, impairment of glucose regulation in WS subjects was characterized by reduction of insulin sensitivity, which prevailed in IGT individuals and preceded β-cell dysfunction, that appeared to drive transition from IGT to IFG+IGT and particularly DM.

### Genes potentially involved in the development of IGM in the WS

Taken together, these findings support the hypothesis that hemizygosity for one or more genes of WSCR is implicated in the abnormalities of glucose regulation which frequently occur in WS patients. Both the candidate genes STX-1A and MLXIPL/ChREBP might be involved in the development of IGM and DM, accounting for β-cell dysfunction and insulin resistance, respectively.

The STX-1A gene encodes for the STX-1A protein, which is involved in β-cell function in vesicles docking and fusion [26] and the release of insulin granules responsible for first-phase insulin secretion [27] by interacting with the nucleotide-binding domains of sulfonylurea receptor 1, thus inhibiting the activity of K_ATP_ channels that couple glucose concentrations to insulin secretion [28]. Studies in STX-1A knockout mice islets showed that this protein is involved in fusion of previously docked granules during first-phase insulin secretion, but not in fusion from newcomers, which are responsible for second-phase release, that was preserved in these animals [29], consistent with findings in WS subjects.

The MLXIPL gene encodes for the basic-helix-loop-helix leucine zipper protein MLXIPL/ChREBP [11], which forms an heterodimeric complex with MLX [11], a member of the Myc-Max family of basic helix–loop–helix transcription factors [30]. This complex translocates into the nucleus, where it regulates the expression of target genes with carbohydrate response element motifs in their promoter [31]. These genes code for enzymes of glycolysis and pentose phosphate pathway as well as of fatty acid synthesis and assembly into triglycerides [32,33]. Therefore, MLXIPL/ChREBP mediates conversion of sugars into lipids [34], as confirmed by studies in ChREBP knockout mice showing elevated circulating glucose and insulin levels and reduced fatty acid and increased glycogen liver content [35], consistent with the low triglyceride levels and normal lipid profile observed in WS patients.

### Strengths and limitations

Strengths of this study include the longitudinal design, with a long follow-up duration; the relatively large sample size, considering the rarity of the WS; and the wide range of indices of insulin secretion and/or sensitivity assessed.

However, though these simple OGTT-based indices were shown to have a good discriminatory ability for predicting IGM and type 2 DM [22], they may be inferior to the more complex “gold standard” measures of insulin secretion and sensitivity obtained by the use of the frequently sampled intravenous glucose tolerance test or the glucose clamp techniques.

### Conclusions

This study demonstrates a high incidence and prevalence of IGM (and DM) in adult WS subjects, thus supporting the need for early screening and continuous follow-up of these individuals with repeated OGTTs. It also indicates the importance of a timed counseling intervention for retarding or reversing these abnormalities. Finally, our data show for the first time that, also in WS subjects, impairment of both insulin secretion and sensitivity contribute to the development of altered glucose regulation, with insulin resistance preceding β-cell dysfunction in the progression from NGF/NGT to IGM and DM and lifestyle exerting a modulatory effect.

## Supporting information

S1 Table Yearly category of glucose homeostasis in each of the 31 study subjects. (DOCX)

S1 Fig Baseline (white bars) and end-of-follow-up (black bars) values of CPGI (a), CIR_120_ (b), HOMA-IR (I) (c), QUICKI (d), MI (e), oDI (I) (f), oDI (CP) (g), and ISSI-2 (h) of WS patients who progressed and those who did not progress from NFG/NGT to IFG and/or IGT or from IGT and/or IFG to DM.
(DOCX)

S1 STROBE Statement Checklist of items for reports of cohort studies. (DOC)
