# Supplementary material for: Impaired glucose metabolism in subjects with the Williams-Beuren syndrome: A five-year follow-up cohort study
Source: PLoS One. 2017 Oct 20;12(10):e0185371. doi: 10.1371/journal.pone.0185371 (PMC5650138; doi:10.1371/journal.pone.0185371)
Supplement: S1 Table — (DOCX) [file pone.0185371.s001.docx]

**S1 Table.** Yearly category of glucose homeostasis in each of the 31 study subjects.

| **Group** | **Subject** | **Baseline** | **Follow-up (year)** | | | | | |
| --- | --- | --- | --- | --- | --- | --- | --- | --- |
|  |  |  | **1** | **2** | **3** | **4** | **5** |  |
| **Progressors** | **1** | NFG/NGT | NFG/NGT | IGT | IFG+IGT | IGT | IFG |  |
|  | **2** | NFG/NGT | IGT | NFG/NGT | NFG/NGT | NFG/NGT | IGT |  |
|  | **3** | NFG/NGT | NFG/NGT | IGT | IGT | IFG+IGT | IFG+IGT |  |
|  | **4** | NFG/NGT | NFG/NGT | NFG/NGT | NFG/NGT | NFG/NGT | IFG+IGT |  |
|  | **5** | NFG/NGT | NFG/NGT | IGT | IGT | IGT | IFG+IGT |  |
|  | **6** | IGT | DM | IGT | DM | DM | DM |  |
|  | **7** | IFG+IGT | IGT | IGT | IGT | IGT | DM |  |
|  | **8** | IFG+IGT | IFG+IGT | DM | DM | DM | DM |  |
| **Regressors** | **9** | IGT | IGT | IGT | IGT | NFG/NGT | NFG/NGT |  |
|  | **10** | IGT | NFG/NGT | NFG/NGT | NFG/NGT | NFG/NGT | NFG/NGT |  |
|  | **11** | IGT | NFG/NGT | NFG/NGT | NFG/NGT | NFG/NGT | NFG/NGT |  |
|  | **12** | IFG+IGT | NFG/NGT | NFG/NGT | NFG/NGT | NFG/NGT | NFG/NGT |  |
|  | **13** | DM | IGT | IGT | IGT | NFG/NGT | IGT |  |
| **Non-progressors** | **14** | NFG/NGT | NFG/NGT | NFG/NGT | NFG/NGT | NFG/NGT | NFG/NGT |  |
|  | **15** | NFG/NGT | NFG/NGT | NFG/NGT | NFG/NGT | NFG/NGT | NFG/NGT |  |
|  | **16** | NFG/NGT | NFG/NGT | NFG/NGT | NFG/NGT | NFG/NGT | NFG/NGT |  |
|  | **17** | NFG/NGT | NFG/NGT | NFG/NGT | NFG/NGT | NFG/NGT | NFG/NGT |  |
|  | **18** | NFG/NGT | NFG/NGT | NFG/NGT | NFG/NGT | NFG/NGT | NFG/NGT |  |
|  | **19** | NFG/NGT | NFG/NGT | NFG/NGT | NFG/NGT | NFG/NGT | NFG/NGT |  |
|  | **20** | NFG/NGT | IGT | IGT | NFG/NGT | NFG/NGT | NFG/NGT |  |
|  | **21** | NFG/NGT | IGT | IGT | IGT | NFG/NGT | NFG/NGT |  |
| **Non-regressors** | **22** | IGT | NFG/NGT | IGT | IGT | IGT | IGT |  |
|  | **23** | IGT | IGT | NFG/NGT | IGT | IGT | IGT |  |
|  | **24** | IGT | IGT | IGT | IGT | IGT | IGT |  |
|  | **25** | IGT | IGT | IGT | IFG+IGT | IFG+IGT | IFG+IGT |  |
|  | **26** | IFG+IGT | IFG+IGT | IGT | IGT | IGT | IGT |  |
|  | **27** | IFG+IGT | DM | IGT | IGT | IGT | IGT |  |
|  | **28** | IFG+IGT | IGT | IGT | IFG+IGT | IGT | IFG+IGT |  |
|  | **29** | IFG+IGT | IFG+IGT | IFG+IGT | IFG+IGT | IFG+IGT | IFG+IGT |  |
|  | **30** | DM | NGT | IGT | IFG+IGT | IFG+IGT | DM |  |
|  | **31** | DM | DM | DM | DM | DM | DM |  |

NFG = normal fasting glucose; NGT = normal glucose tolerance; IFG = impaired fasting glucose; IGT = impaired glucose tolerance; DM = diabetes mellitus.
