# Supplementary material for: Impaired glucose metabolism in subjects with the Williams-Beuren syndrome: A five-year follow-up cohort study
Source: PLoS One. 2017 Oct 20;12(10):e0185371. doi: 10.1371/journal.pone.0185371 (PMC5650138; doi:10.1371/journal.pone.0185371)

**S1 Fig.** Baseline (white bars) and end-of-follow-up (black bars) values of CPGI (a), CIR_120_ (b), HOMA-IR (I) (c), QUICKI (d), MI (e), oDI (I) (f), oDI (CP) (g), and ISSI-2 (h) of WS patients who progressed and those who did not progress from NFG/NGT to IFG and/or IGT or from IGT and/or IFG to DM.

*P* by paired Student’s t test. CPGI = C-peptidogenic Index; CIR = Corrected Insulin Response; HOMA-IR = Homeostasis Model Assessment-Insulin Resistance; I = insulin; QUICKI = Quantitative Insulin Check Index; MI = Composite Insulin Sensitivity Index or Matsuda Index; oDI = oral Disposition Index; CP = C-peptide; ISSI-2 = Insulin Secretion-Sensitivity Index-2; NFG = normal fasting glucose; NGT = normal glucose tolerance; IFG = impaired fasting glucose; IGT = impaired glucose tolerance; DM = diabetes mellitus.


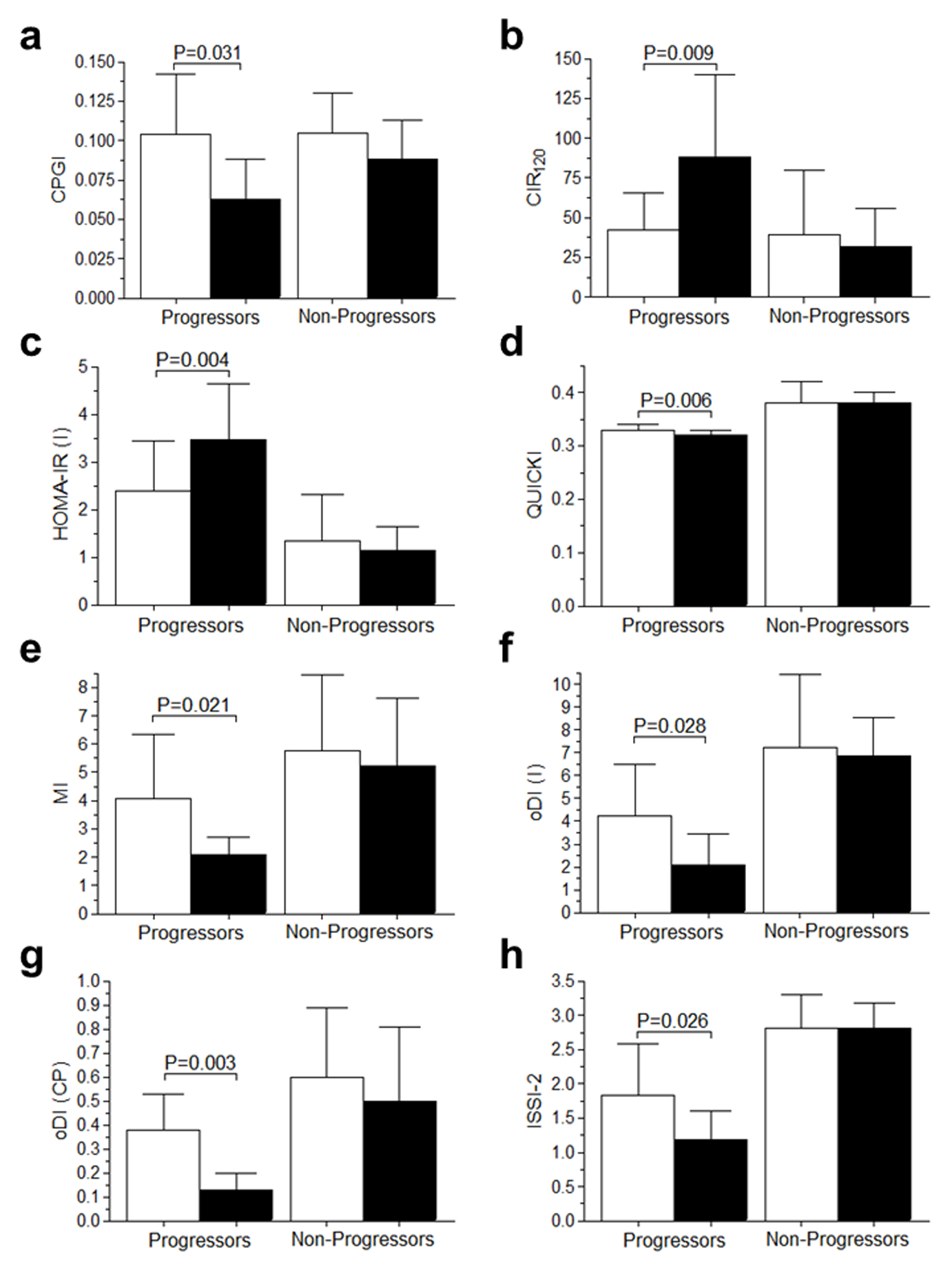

Supplement: S1 Fig — Baseline (white bars) and end-of-follow-up (black bars) values of CPGI (a), CIR120 (b), HOMA-IR (I) (c), QUICKI (d), MI (e), oDI (I) (f), oDI (CP) (g), and ISSI-2 (h) of WS patients who progressed and those who did not progress from NFG/NGT to IFG and/or IGT or from IGT and/or IFG to DM. (DOCX) [file pone.0185371.s002.docx]
